# SADI-S and SG surgeries induce distinct bile acid profiles linked to improved glucose metabolism via microbiota interactions

**DOI:** 10.3389/fmicb.2025.1579149

**Published:** 2025-10-02

**Authors:** Subo Ma, Zheng Zhang, Zhiqiang Wei, Lifu Hu, Lun Wang, Zhubin Shen, Chaonv Fang, Tao Jiang

**Affiliations:** ^1^Department of Bariatric and Metabolic Surgery, China-Japan Union Hospital of Jilin University, Changchun, China; ^2^Department of Gastrointestinal Surgery, Affiliated Hospital of Zunyi Medical University, Zunyi, China; ^3^Department of Radiology, China-Japan Union Hospital of Jilin University, Changchun, China; ^4^Department of Hepatobiliary and Pancreatic Surgery, The First Affiliated Hospital, Zhejiang University School of Medicine, Hangzhou, China

**Keywords:** single-anastomosis duodenal-ileal bypass with sleeve gastrectomy, sleeve gastrectomy, bile acid, gut microbiota, T2D

## Abstract

**Introduction:**

Although bariatric surgery profoundly ameliorates type 2 diabetes (T2D), the mechanisms whereby specific procedures confer metabolic benefits through bile acid (BA) remodeling remain incompletely defined. This study compared the effects of single-anastomosis duodenal-ileal bypass with sleeve gastrectomy (SADI-S) and sleeve gastrectomy (SG) on BA profiles and their association with metabolic outcomes in a rodent model of T2D.

**Methods:**

Male Wistar rats with T2D underwent SADI-S, SG, or sham operation. Metabolic parameters, including fasting blood glucose, HbA1c, glucagon-like peptide-1 levels, triglycerides, gut microbiota composition, and comprehensive serum/fecal BA profiles, were assessed 5 weeks post-surgery. Statistical analyses included t-tests and Pearson correlations, with false discovery rate correction applied.

**Results:**

Both SADI-S and SG significantly ameliorated hyperglycemia, dyslipidemia, and β-cell integrity compared to sham operation, with SADI-S demonstrating superior efficacy. SADI-S induced a more pronounced elevation of portal serum BAs (34 vs. 25 species in SG), including key regulators such as chenodeoxycholic acid and lithocholic acid. Critically, multiple elevated serum BAs (e.g., chenodeoxycholic acid, lithocholic acid, glycoursodeoxycholic acid) exhibited strong negative correlations with fasting blood glucose, HbA1c, and triglycerides, while positively correlating with glucagon-like peptide-1 levels. Shifts in gut microbiota correlated with specific BA changes, supporting a ‘microbiota-BA-metabolism’ axis.

**Conclusion:**

SADI-S and SG induce distinct, surgery-specific BA remodeling that is significantly associated with metabolic improvements in T2D. The robust correlations between specific BA species and metabolic parameters underscore their potential as mediators and therapeutic targets. SADI-S promotes a more extensive and beneficial BA profile, aligning with its superior metabolic efficacy.

## Introduction

According to the International Diabetes Federation, diabetes represents one of the most rapidly escalating global health emergencies of the 21st century. In 2021, an estimated 537 million adults aged 20 to 79 years were living with diabetes worldwide, a figure projected to rise to 643 million by 2030 and 783 million by 2045. Type 2 diabetes (T2D) is the most prevalent form, accounting for over 90% of total diabetes cases globally (International Diabetes Federation, 2021). T2D and its complications pose a substantial burden on patients’ quality of life and healthcare systems, constituting a major global socioeconomic challenge (Cole and Florez, 2020).

Traditional therapeutic strategies for T2D, which include lifestyle modifications such as diet and exercise, as well as pharmacotherapy, frequently fall short in achieving sustained glycemic control and preventing complications in a considerable proportion of patients. In contrast, metabolic surgery has emerged as a highly effective intervention for T2D, demonstrating superior glycemic outcomes and higher rates of disease remission compared to intensive medical management alone (Mingrone et al., 2021; Schauer et al., 2012).

Currently, metabolic surgeries primarily include Roux-en-Y gastric bypass (RYGB), sleeve gastrectomy (SG), duodenal switch biliopancreatic diversion (BPD-DS), and single-anastomosis duodenal-ileal bypass with sleeve gastrectomy (SADI-S). Among these, RYGB and SG are the most commonly performed procedures. Although BPD-DS is highly effective in treating T2D, its widespread adoption is limited due to associated surgical risks and a higher likelihood of malnutrition. In response to these concerns, SADI-S was proposed in 2007 as an alternative to traditional BPD-DS surgery (Sánchez-Pernaute et al., 2007). SADI-S not only offers similar benefits in alleviating metabolic diseases as BPD/DS but also reduces the number of anastomoses and extends the length of the common intestinal passage, thereby lowering the risks of surgery and malnutrition.

Beyond anatomical and hormonal changes, alterations in bile acid (BA) metabolism are increasingly recognized as key mediators of the metabolic benefits following bariatric surgery. Early work established the crucial role of BAs in regulating lipid metabolism (Angelin et al., 1978). It is now evident that dysregulation of BA signaling is implicated in the pathogenesis of conditions such as severe obesity, insulin resistance, non-alcoholic fatty liver disease, and T2D (Chávez-Talavera et al., 2017). Interventions targeting BA sequestration or BA receptor binding have demonstrated potential for improving glucose homeostasis (Prawitt et al., 2014; Shima et al., 2018). Notably, both clinical and preclinical studies indicate that bariatric surgery profoundly remodels BA metabolism, transport, and receptor signaling, significantly contributing to T2D remission (Cole et al., 2015; Ryan et al., 2014).

Current research on BA remodeling following bariatric surgery has predominantly focused on RYGB and SG. However, the specific alterations in the BA profile induced by SADI-S and how these changes mechanistically link to its metabolic outcomes, particularly glucose metabolism, remain inadequately understood. Our preliminary work has demonstrated significant shifts in BA levels following SADI-S in a rodent model (Wang et al., 2022); nevertheless, the precise relationship between SADI-S-specific BA remodeling and the observed metabolic improvements, especially in comparison to established procedures like SG, represents a notable gap in the literature. Consequently, this study aims to investigate and compare the BA profiles induced by SADI-S and SG surgeries, elucidating their correlations with host metabolic parameters, particularly glucose metabolism. By delineating the distinct BA signatures associated with each procedure and their metabolic implications, we seek to enhance the understanding of the mechanisms underlying the efficacy of SADI-S and to identify potential targets for less invasive therapeutic strategies.

## Materials and methods

### T2D animal model and groups

In this study, 30 male Wistar rats aged 8 weeks (purchased from Beijing Vital River Experimental Animal Technology Co., Ltd.) were selected. T2D was induced as previously described (Wang et al., 2022). Throughout the experiment, all rats were housed individually in a controlled environment with regulated temperature and humidity, following a 12-h light/12-h dark cycle. To maximize control over variables and ensure consistency in experimental conditions, all rats were provided with a specific formulated diet: standard feed for T2D model induction and subsequent experiments (fat 13.8%, carbohydrates 63.4%, protein 22.8%) and high-fat feed (fat 45.6%, carbohydrates 37.9%, protein 16.5%). During the entire study period, the rats in each group strictly adhered to their designated dietary regimen to eliminate potential confounding effects due to dietary differences. After successfully inducing T2D, the 30 rats were randomly divided into three groups: SADI-S group, the SG group, and the sham operation (SO) group, and underwent the corresponding surgical interventions. Postoperatively, the rats in each group continued to be fed their standardized diet to ensure consistency in feeding conditions, thereby ensuring comparability of results between groups. All animal protocols received approval from the Animal Experimentation Ethics Committee of the First Hospital of Jilin University, and the care of the rats adhered to the National Animal Care Guidelines of the People’s Republic of China.

### Surgical procedures

SADI-S: The SADI-S surgery was conducted in accordance with the protocol established by Wang et al. (2022). In summary, two-thirds of the stomach was excised. Subsequently, the duodenum was transected approximately 5 mm from the pylorus, and the duodenal stump was sutured using a 6–0 PDS suture (Ethicon). Following this, a 40-cm segment of the small intestine was measured retrogradely from the ileocecal junction and marked with sutures. An end-to-side anastomosis was then performed between the proximal duodenum and the marked ileal segment, positioned 40 cm from the ileocecal junction. Finally, the abdomen was closed with a 4–0 non-absorbable silk suture. In the sham operation group, rats underwent laparotomy only, with the procedure details based on our previous study (Wang et al., 2022).

SG: In brief, first open the abdominal cavity and then remove two-thirds of the stomach.

### Metabolic evaluation and biochemical analysis

The body weight and blood glucose levels of the rats were recorded before surgery and weekly thereafter. The intraperitoneal glucose tolerance test (IPGTT) and insulin tolerance test (ITT) were conducted by puncturing the tail vein to measure blood glucose levels. In the IPGTT experiment, following a 12-h fasting period, rats were injected intraperitoneally with 2 g/kg of a 20% glucose solution. Blood glucose levels were measured at 0, 15, 30, 60, 120, and 180 min post-glucose challenge (Stable + Code, stable +, Sinocare). The ITT experiment involved an intraperitoneal injection of insulin (0.2 IU/kg) after a 12-h fasting period, with blood glucose levels measured at 0, 15, 30, 60, and 120 min following the insulin challenge. The area under the curve (AUC) at the respective time points of blood sample collection was calculated for both the IPGTT and ITT.

### Glucagon-like peptide-1 (GLP-1) measurement

Plasma active GLP-1 was measured using a rat-specific ELISA kit (Enzyme Immuno Biological, cat# MM-0033R2). Blood was collected in EDTA tubes with a DPP-4 inhibitor 15 min after glucose gavage. Plasma was separated by centrifugation (2,000 × *g*, 15 min, 4°C) and stored at −80°C. Samples were diluted 5-fold and analyzed in duplicate per the manufacturer’s protocol. Absorbance was read at 450 nm (USCNK SMR60047). The detection range was 0.15–4 pmol/L. Concentrations were calculated from a standard curve and reported in pmol/L.

### Histological evaluation

Five weeks post-operation, rat pancreatic tissue samples were fixed in paraformaldehyde solution, embedded, and sectioned into 4-micron-thick slices. Immunofluorescent double-labeling was then employed for staining. The pathological changes in the pancreas were subsequently observed using a fluorescence microscope.

### Gut microbiota analysis

See Supplementary Material 1.

### Plasma and fecal BA measurements

See Supplementary Material 2.

### Statistical analysis

*A priori* comparisons were conducted using unpaired t-tests (GraphPad Prism 9.5.1) to compare each bariatric surgery group (SG and SADI-S) against the SO group, as well as to compare SADI-S directly with SO. Additionally, Pearson correlation analysis (GraphPad Prism 9.5.1) was performed to assess the relationships between BA, metabolic variables, and gut microbiota. Normality of data for correlation analysis was assessed using the Shapiro–Wilk test. As the data met the assumption of normality (*p* > 0.05), Pearson correlation analysis was employed. The robustness of the correlations was confirmed by parallel non-parametric Spearman analysis, which yielded consistent results. Given the potential for multiple comparisons in the correlation analysis, the Benjamini-Hochberg correction was applied to control the false discovery rate (FDR) at a significance level of 0.05. This approach effectively balances the control of false positives with the maintenance of statistical power. Correlation analyses included only animals with complete datasets across all measured parameters (BA profiles, metabolic variables, microbiota). All data are expressed as Mean ± SEM.

## Results

### Surgical outcome

In the SADI-S group, two rats died due to anastomotic leakage, and one rat died from bleeding. Ultimately, seven rats survived for 5 weeks following SADI-S surgery. In the SG group, one rat died from hemorrhage, and one rat died from anastomotic leakage. In total, eight rats in this group survived to 5 weeks post-surgery. Three rats died due to incision healing complications. It was hypothesized that the failure of the incision to heal was attributed to persistent hyperglycemia. Notably, the last seven rats survived for 5 weeks post-surgery.

### Changes in body weight and metabolism

**Figure 1:**
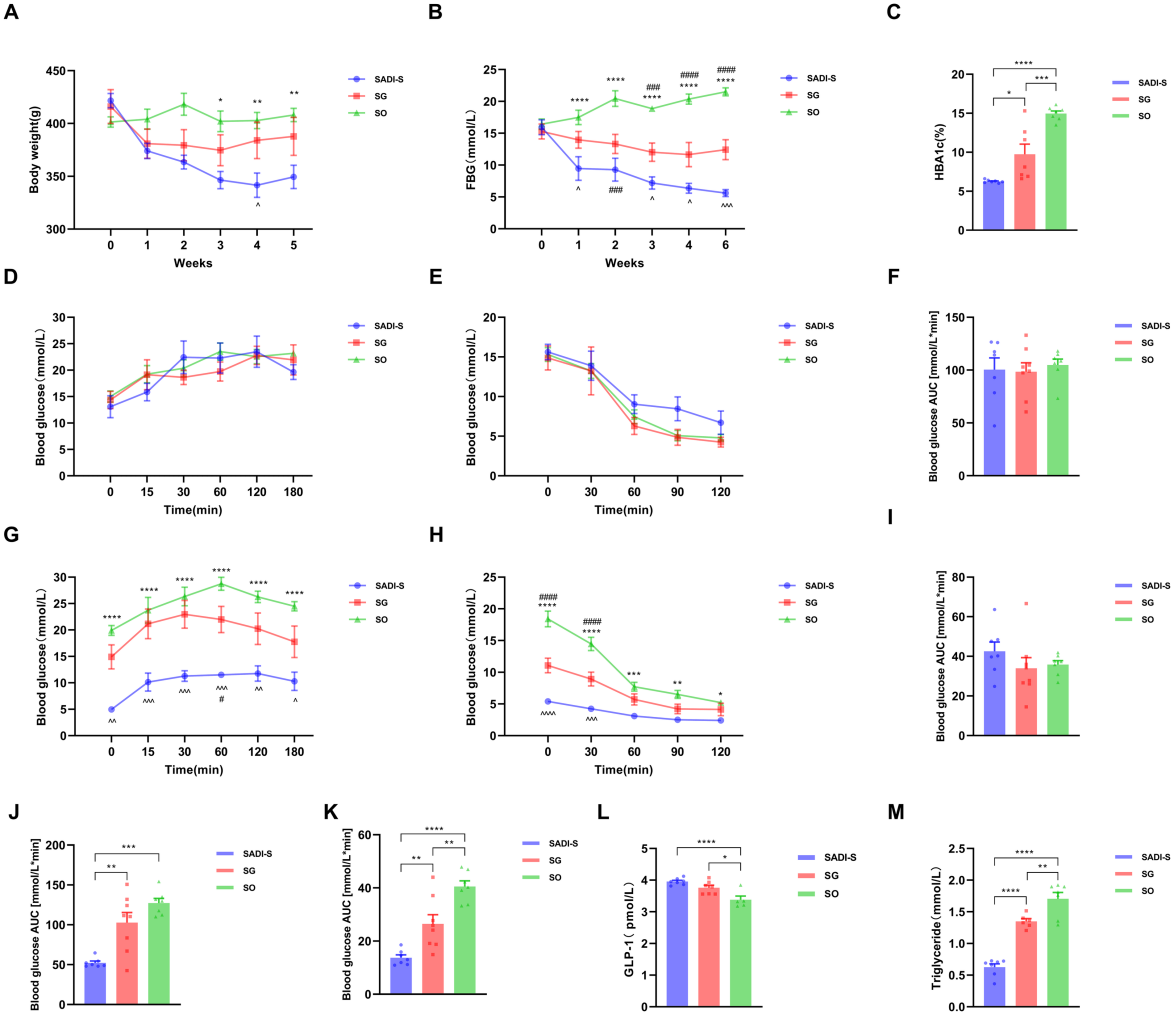
Variations in body weight and metabolic indicators across three groups of diabetic rats. **(A)** Body weight alterations. **(B)** FBG changes. **(C)** HbA1c levels observed in each group 5 weeks post-surgery. **(D)** IPGTT levels before the operation. **(E)** ITT levels before the operation. **(F)** AUC for preoperative IPGTT. **(G)** IPGTT levels after surgery. **(H)** ITT levels following surgery. **(I)** AUC for preoperative ITT. **(J)** AUC for postoperative IPGTT. **(K)** AUC for postoperative ITT. **(L)** Serum levels of GLP-1 15 min after glucose administration in each group. **(M)** Triglyceride levels across each group. Sample sizes: SADI-S (*n* = 7), SG (*n* = 8), SO (*n* = 7). SADI-S, Single-anastomosis duodenal-ileal bypass with sleeve gastrectomy; SG, Sleeve gastrectomy; SO, Sham operation; FBG, Fasting blood glucose; HbA1c, Glycated hemoglobin; IPGTT, Intraperitoneal glucose tolerance test; ITT, Insulin tolerance test; AUC, Area under the curve; GLP-1, glucagon-like peptide-1. Unmarked means no statistically significant difference between groups. Data are mean ± SEM. *, SADI-S vs. SO; ^, SADI-S vs. SG; #, SG vs. SO. */#/^*p* < 0.05, **/##/^^*p* < 0.01; ***/###/^^^*p* < 0.001; ****/####/^^^^*p* < 0.0001.

Fasting blood glucose (FBG) and body weight did not differ significantly between groups before surgery. After the procedure, both body weight and FBG levels in the SADI-S and SG groups gradually decreased compared to the SO group, with a more pronounced decrease observed in the SADI-S group (Figures 1A,B). The levels of glycated hemoglobin (HbA1c) in the SADI-S and SG groups were significantly lower than those in the SO group (*p* < 0.05). Furthermore, HbA1c levels in the SADI-S group were also significantly lower than those in the SG group (*p* < 0.05) (Figure 1C). No significant differences were noted in IPGTT and ITT blood glucose levels, as well as their corresponding AUC, at any time point between the groups before surgery (Figures 1D–F,I). Five weeks post-surgery, the blood glucose levels and AUC at each time point for IPGTT and ITT in the SADI-S group were significantly lower than those in the SO group (*p* < 0.05) (Figures 1G,H,J,K). Additionally, GLP-1 levels in both the SADI-S and SG groups were significantly higher than those in the SO group (*p* < 0.05). Triglyceride levels were significantly lower in the SADI-S and SG groups than in the SO group (*p* < 0.05, Figure 1M).

### Histological assessment of the pancreas

**Figure 2:**
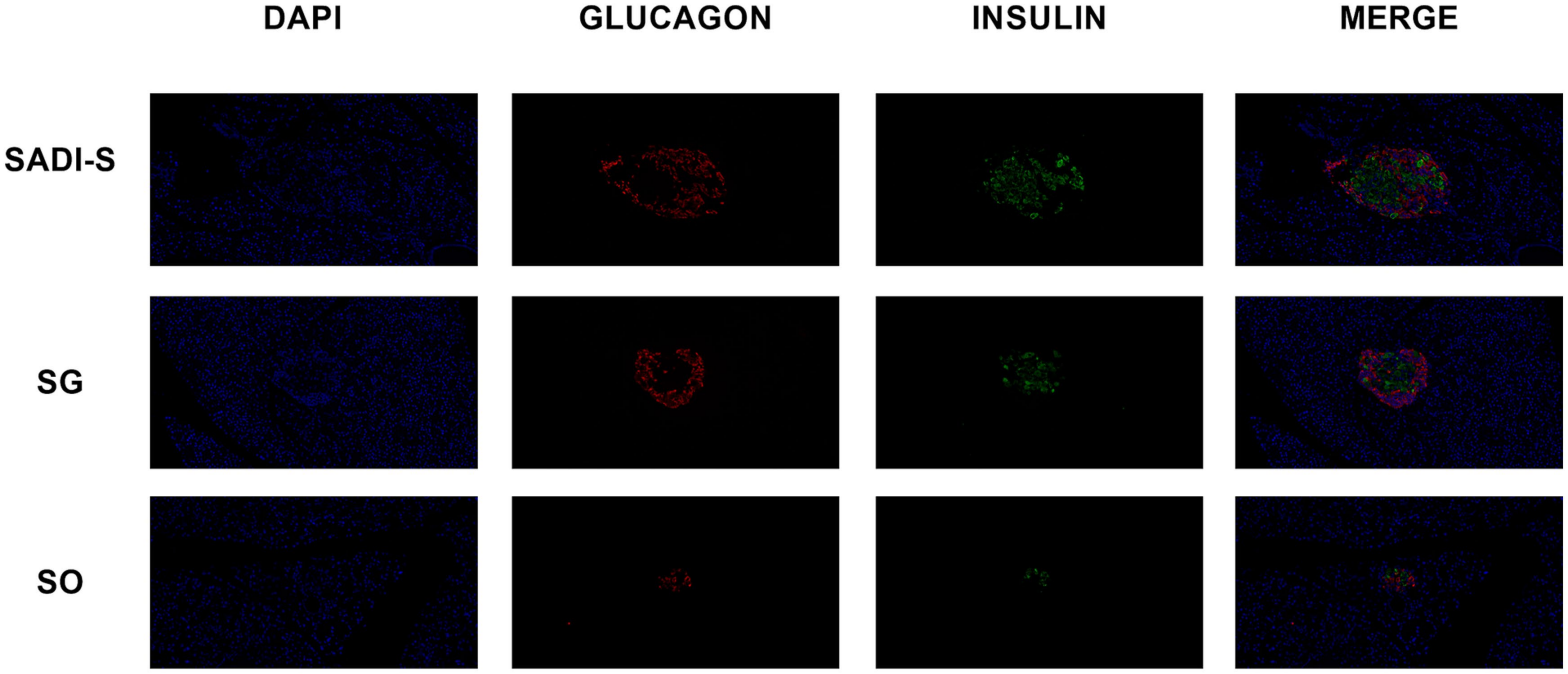
Immunofluorescence double-labeling staining images of the pancreas were obtained from the SADI-S group, as well as the SG and SO groups. In these images, red indicates pancreatic islet *α* cells, green indicates pancreatic islet β cells, and blue represents the nucleus. SADI-S, Single-anastomosis duodenal-ileal bypass with sleeve gastrectomy; SG, Sleeve gastrectomy; SO, Sham operation.

Figure 2 presents the immunofluorescence double-labeling staining image of pancreatic tissue. In comparison to the SADI-S and SG groups, the SO group exhibited a significant reduction in the number of islet β cells, accompanied by notable vacuolar degeneration. Additionally, the SADI-S and SG groups demonstrated a uniform distribution pattern and morphology of β cells when compared to the SO group.

### Intestinal microbial species distribution histogram

**Figure 3:**
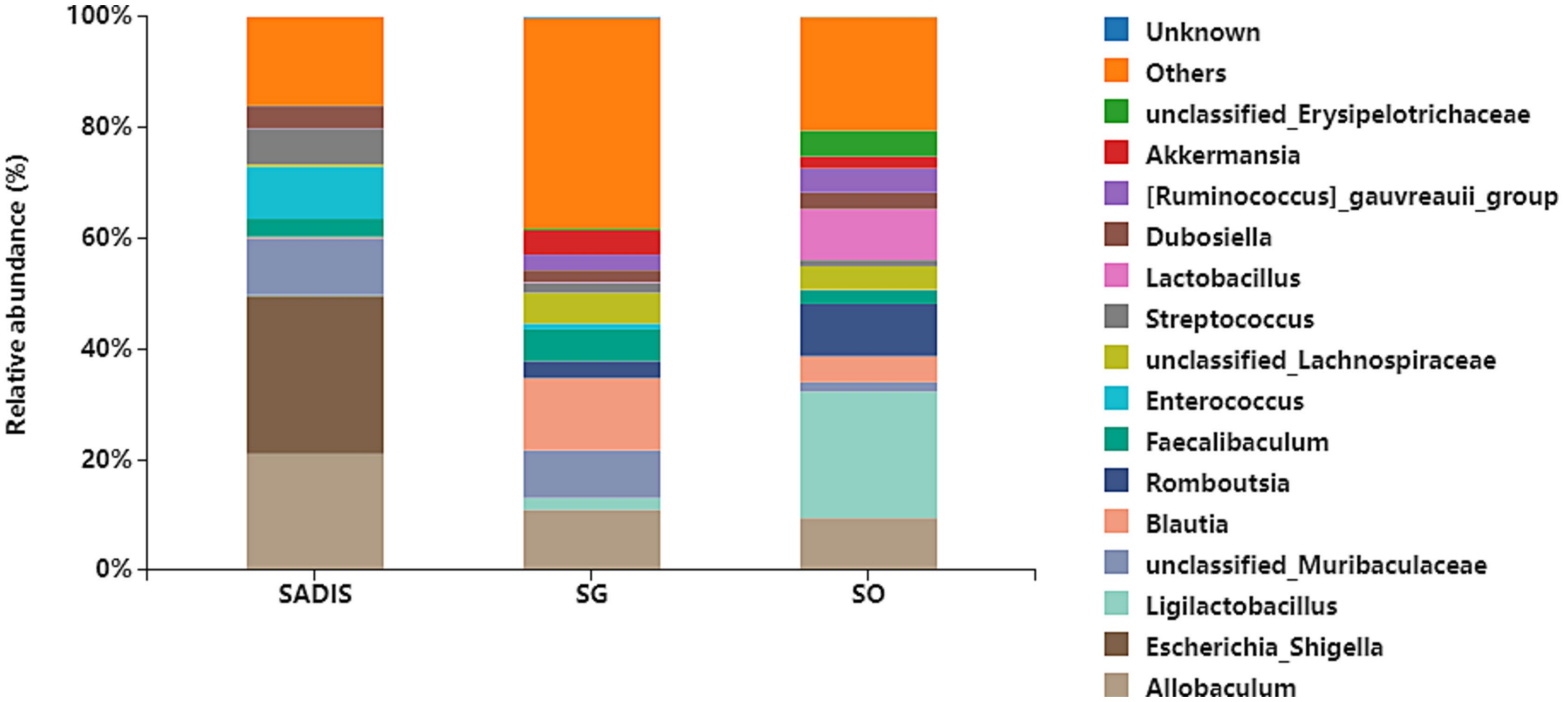
Taxonomic composition of intestinal microbiota at each group’s genus level. Sample sizes: SADI-S (*n* = 6), SG (*n* = 5), SO (*n* = 4). SADI-S, Single-anastomosis duodenal-ileal bypass with sleeve gastrectomy; SG, Sleeve gastrectomy; SO, Sham operation.

Figure 3 shows the taxonomic composition of the intestinal microbiota at the genus level in each group. The 15 most abundant genera were *Allobaculum, Escherichia-Shigella, Ligilactobacillus, unclassified_Muribaculaceae, Blautia, Romboutsia, Faecalibaculum, Enterococcus, unclassified_Lachnospiraceae, Streptococcus, Lactobacillus, Dubosiella, [Ruminococcus]_gauvreauii_group, Akkermansia* and *unclassified_Erysipelotrichaceae*.

### Serum and fecal BA levels after metabolic surgery

**Figure 4:**
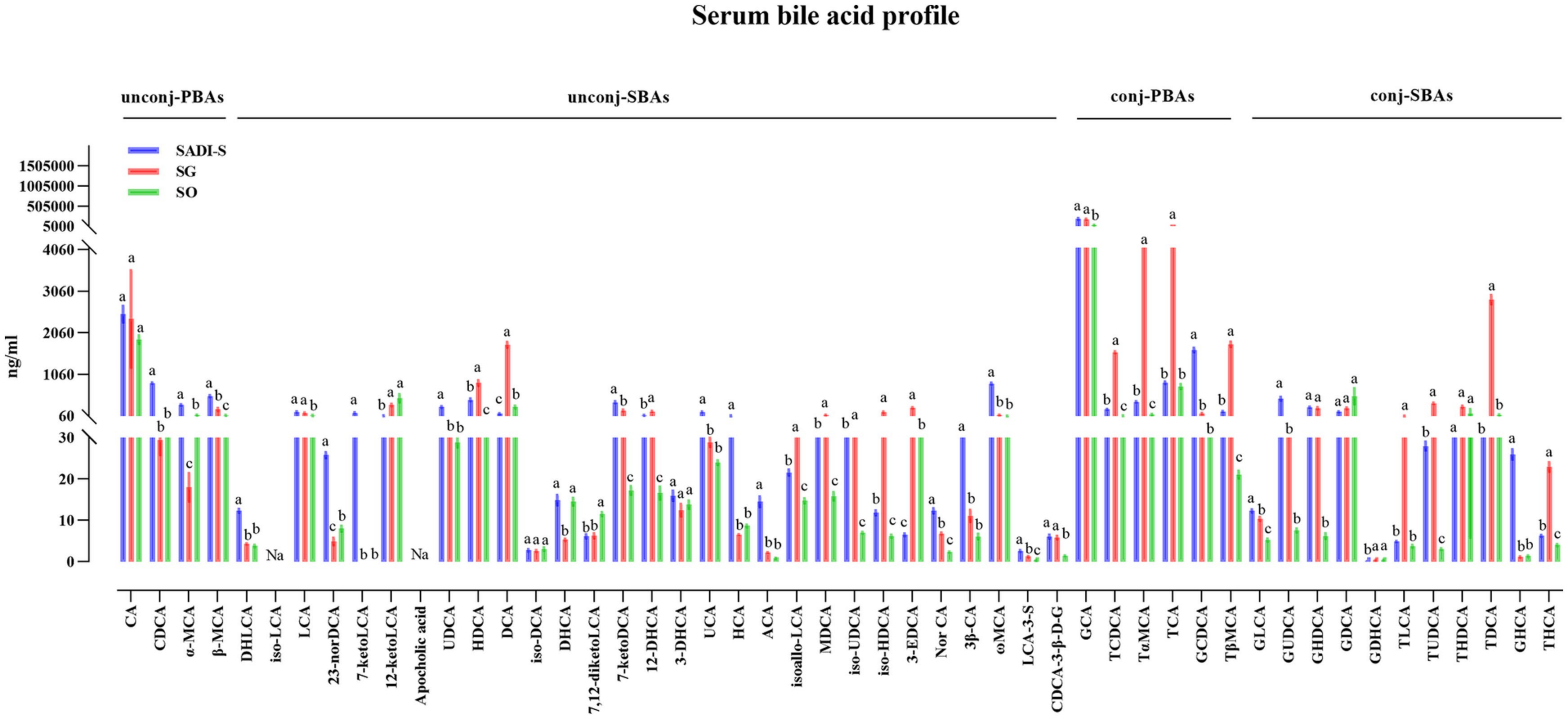
The comparison of portal BAs in T2D rats after SADI-S and SG at 5 weeks post-operation. Sample sizes: SADI-S (*n* = 6), SG (*n* = 3), SO (*n* = 5). SADI-S, single-anastomosis duodenal-ileal bypass with sleeve gastrectomy; SG, sleeve gastrectomy; SO, Sham operation; unconj-PBAs, unconjugated primary bile acids; unconj-SBAs, unconjugated secondary bile acids; conj-PBAs, conjugated primary bile acids; conj-SBAs, conjugated secondary bile acids. For the full names of bile acids, see Supplementary Material 3. Data are mean ± SEM. If two groups have the same letter, the difference between them is not significant; while groups marked with different letters indicate that they are significantly different (*p* < 0.05).

**Figure 5:**
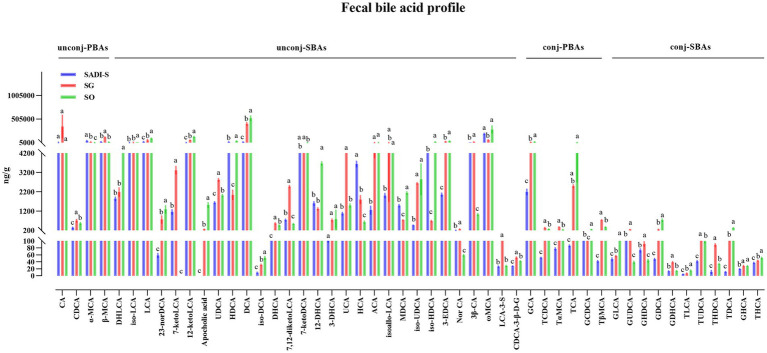
Five weeks post-surgery, the fecal BA profile analysis of T2D rats was conducted following SADI-S and SG procedures. Sample sizes: SADI-S (*n* = 6), SG (*n* = 5), SO (*n* = 5). SADI-S, single-anastomosis duodenal-ileal bypass with sleeve gastrectomy; SG, sleeve gastrectomy; SO, Sham operation; unconj-PBAs, unconjugated primary bile acids; unconj-SBAs, unconjugated secondary bile acids; conj-PBAs, conjugated primary bile acids; conj-SBAs, conjugated secondary bile acids. For the full names of bile acids, see Supplementary Material 3. Data are mean ± SEM. If two groups have the same letter, the difference between them is not significant, while groups marked with different letters indicate that they are significantly different (*p* < 0.05).

**Figure 6:**
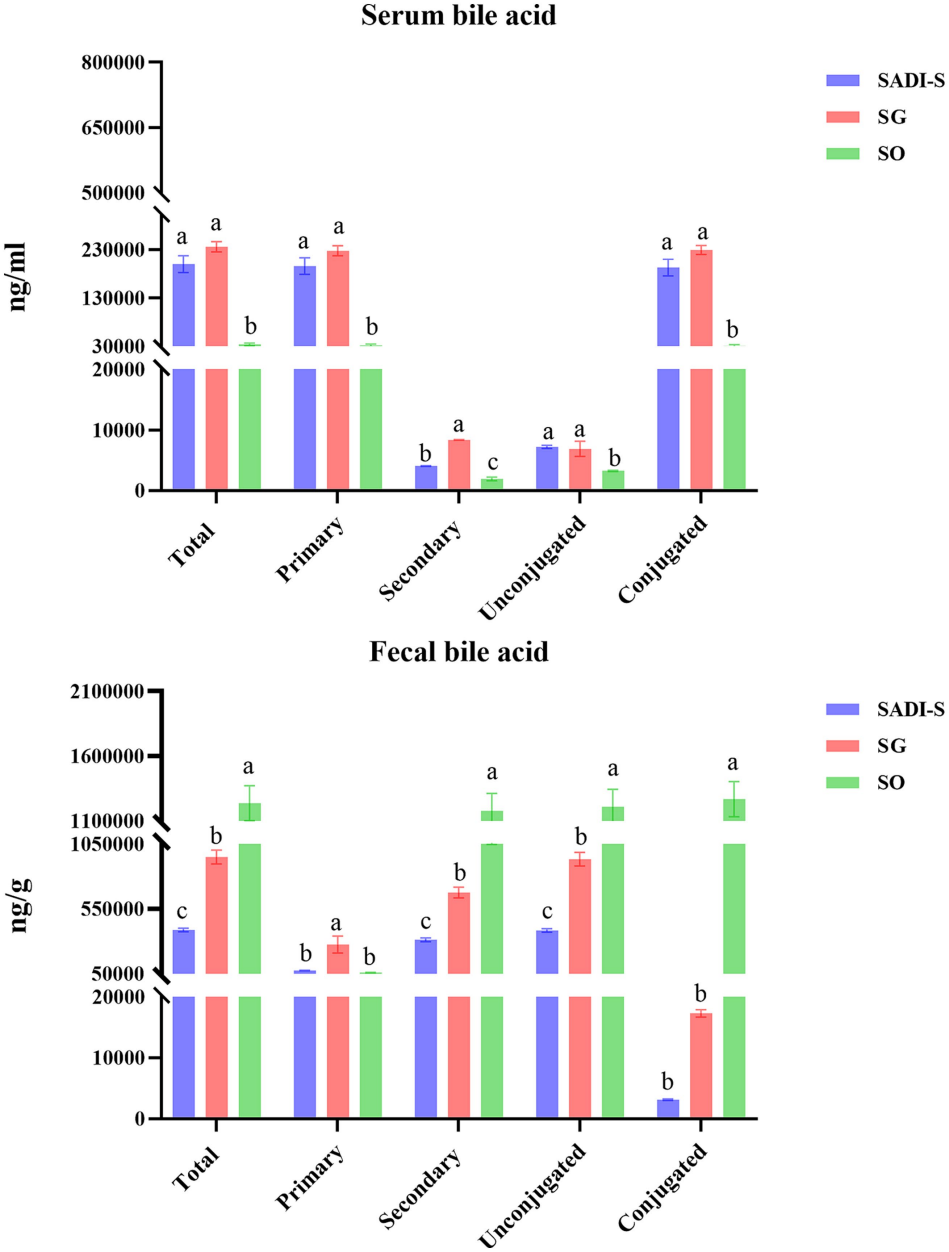
Classification of three different groups of BAs. Sample sizes as in Figures 4, 5. SADI-S, single-anastomosis duodenal-ileal bypass with sleeve gastrectomy; SG, sleeve gastrectomy; SO, Sham operation; Data are mean ± SEM. If two groups have the same letter, the difference between them is not significant, while groups marked with different letters indicate that they are significantly different (*p* < 0.05).

Compared to the SO group, the SADI-S group exhibited significant increases in portal serum BAs, including chenodeoxycholic acid (CDCA), α-Muricholic acid (α-MCA), *β*-Muricholic acid (β-MCA), dehydrolithocholic acid (DHLCA), lithocholic acid (LCA), 23-nordeoxycholic acid (23-norDCA), 7-ketolithocholic acid (7-ketoLCA), ursodeoxycholic acid (UDCA), hyodeoxycholic acid (HDCA), 7-ketodeoxycholic acid (7-ketoDCA), 12-dehydrocholic acid (12-DHCA), ursocholic acid (UCA), hyocholic acid (HCA), allocholic acid (ACA), isoallolithocholic acid, (isoallo-LCA), murideoxycholic acid (MDCA), isoursodeoxycholic acid (iso-UDCA), isohyodeoxycholic acid (iso-HDCA), nor cholic acid (Nor CA), 3β-cholic acid (3β-CA), ω-muricholic acid (ω-MCA), lithocholic acid-3-sulfate (LCA-3-S), CDCA-3-β-D-glucuronide (CDCA-3-β-D-G), glycocholic acid (GCA), taurochenodeoxycholic acid (TCDCA), tauro α-muricholic acid (TαMCA), glycochenodeoxycholic acid (GCDCA), tauro β-muricholic acid (TβMCA), glycolithocholic acid (GLCA), glycoursodeoxycholic acid (GUDCA), glycohyodeoxycholic acid (GHDCA), tauroursodeoxycholic acid (TUDCA), glycohyocholic acid (GHCA), and taurohyocholic acid (THCA). Conversely, the SG group demonstrated significant increases in β-MCA, LCA, HDCA, deoxycholic acid (DCA), 7-ketoDCA, 12-DHCA, isoallo-LCA, MDCA, iso-UDCA, iso-HDCA, 3-dehydrocholic acid (3-EDCA), Nor CA, LCA-3-S, CDCA-3-β-D-G, GCA, TCDCA, TαMCA, taurocholic acid (TCA), TβMCA, GLCA, GHDCA, taurolithocholic acid (TLCA), TUDCA, taurodeoxycholic acid (TDCA), and THCA (Figure 4). In terms of fecal BAs, most BAs were significantly lower in the SADI-S group compared with the SG and SO groups (Figure 5). The total bile acids (TBAs), primary bile acids (PBAs), secondary bile acids (SBAs), unconjugated bile acids (unconj-BAs), and conjugated bile acids (conj-BAs) in the serum of the SADI-S and SG groups were significantly higher than those in the SO group. Additionally, TBAs, SBAs, unconj-BAs, and conj-BAs in feces were significantly lower in the SADI-S and SG groups compared to the SO group (Figure 6).

### Relationships between serum BAs and metabolic variables and the relative abundance of bacterial genera

**Figure 7:**
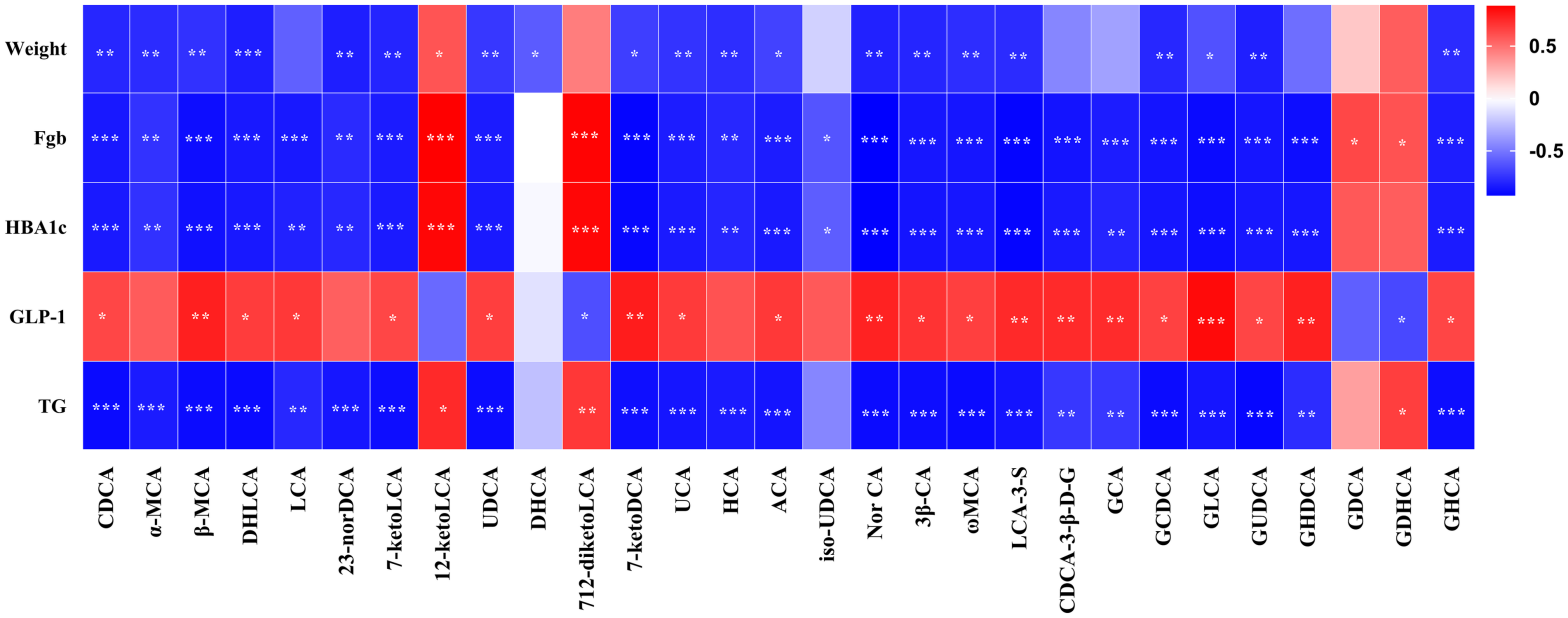
Pearson’s correlations between serum BAs and metabolic variables. Total samples: *n* = 14 rats with complete BA and metabolic data. FBG, fasting blood glucose; HbA1c, glycated hemoglobin; GLP-1, glucagon-like peptide-1; TG, triglyceride. For the full names of bile acids, see Supplementary Material 3. *p < 0.05, **p < 0.01, ***p < 0.001.

**Figure 8:**
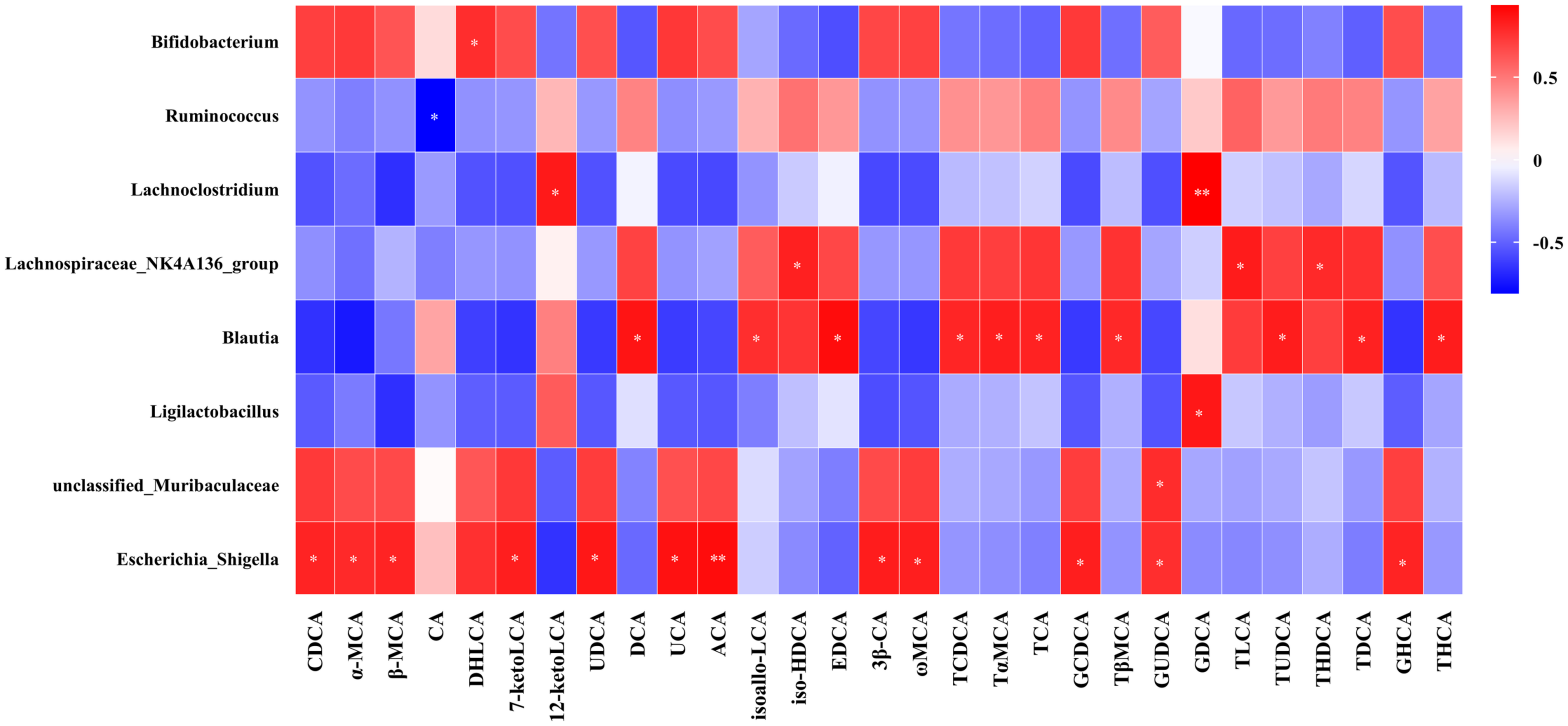
Pearson’s correlations between serum BAs and the abundance of bacterial genera. Total samples: *n* = 13 rats with complete BA and microbiota data. For the full names of bile acids, see Supplementary Material 3. *p < 0.05, **p < 0.01, ***p < 0.001.

Figure 7 presents the Spearman rank correlation analysis between weight and metabolic indicators with serum BAs in the portal vein at 5 weeks post-operation, displaying only those with statistical significance. Multiple BAs, including CDCA, α-MCA, β-MCA, DHLCA, LCA, 23-norDCA, 7-ketoLCA, UDCA, 7-ketoDCA, UCA, HCA, ACA, Nor CA, 3β-CA, ω-MCA, LCA-3-S, CDCA-3-β-D-G, GCA, GCDCA, GLCA, GUDCA, GHDCA, and GHCA were found to have a negative correlation with FBG, HbA1c, and triglycerides, with the exceptions of 12-ketolithocholic acid (12-ketoLCA) and 7,12-diketolithocholic acid (7,12-diketoLCA), which were positively correlated. Most of these (except for LCA, CDCA-3-β-D-G, GCA, and GHDCA) were also negatively correlated with body weight, while the majority (except for α-MCA, 23-norDCA, and HCA) showed a positive correlation with GLP-1. In addition, Figure 8 displays the Spearman rank correlation analysis of the top 20 abundant gut bacterial genera with 50 types of BAs, only presenting statistically significant results. The findings are as follows: *Bifidobacterium* showed a significant positive correlation with DHLCA; *Ruminococcus* exhibited a significant negative correlation with cholic acid (CA); *Lachnoclostridium* demonstrated a significant positive correlation with 12-ketoLCA and glycodeoxycholic acid (GDCA); *Lachnospiraceae_NK4A136_group* was significantly positively correlated with iso-HDCA, TLCA, and taurohyodeoxycholic acid (THDCA); *Blautia* displayed significant positive correlations with DCA, isoallo-LCA, EDCA, TCDCA, T*α*MCA, TCA, T*β*MCA, TUDCA, TDCA, and THCA; *Ligilactobacillus* was significantly positively correlated with GDCA; *unclassified_Muribaculaceae* showed a significant positive correlation with GUDCA; *Escherichia-Shigella* exhibited substantial positive correlations with CDCA, α-MCA, β-MCA, 7-ketoLCA, UDCA, UCA, ACA, 3β-CA, *ω*-MCA, GCDCA, GUDCA, and GHCA.

## Discussion

Alterations in BAs following metabolic surgery contribute significantly to T2D improvement (Cole et al., 2015; Ryan et al., 2014). Our preliminary work also indicated marked changes in BAs after SADI-S (Wang et al., 2022). However, the specific relationship between distinct BA remodeling induced by different procedures, particularly SADI-S versus the established SG, and the resultant metabolic benefits, especially regarding glucose metabolism, remains incompletely defined. This study aims to delineate the BA profiles specific to SADI-S and SG surgeries and elucidate their correlations with host metabolic parameters. Our findings reveal that both procedures induce distinct, surgery-specific BA remodeling, which is robustly correlated with metabolic improvements. Crucially, SADI-S elicits a more extensive and beneficial BA profile compared to SG, aligning with its superior metabolic efficacy. To provide a clearer mechanistic framework, we discuss these findings by focusing on three interconnected axes: BA signaling pathways, GLP-1 modulation, and microbiota interactions.

Our results demonstrate profound and differential BA remodeling following SADI-S and SG. Both SADI-S and SG significantly elevated portal serum BAs compared to SO, with SADI-S showing a broader spectrum of elevated species (34 vs. 25 in SG), encompassing key regulators such as CDCA, LCA, α, β, ω-MCA, and GUDCA (Figure 4). Notably, numerous elevated BAs, including CDCA, LCA, α-MCA, β-MCA, DHLCA, and GUDCA, exhibited strong negative correlations with FBG, HbA1c, and triglycerides (Figure 7). This pattern aligns with the established roles of specific BAs as ligands for receptors central to metabolic regulation. CDCA, prominently elevated in SADI-S, is a potent dual activator of the farnesoid X receptor (FXR) and Takeda G protein-coupled receptor 5 (TGR5) (Konstantinovna and Vishal, 2024; Wu et al., 2023). FXR activation enhances insulin sensitivity, suppresses hepatic gluconeogenesis, and modulates lipid metabolism. Concurrently, TGR5 agonism directly stimulates GLP-1 secretion from intestinal L cells (Konstantinovna and Vishal, 2024; Wu et al., 2023). LCA, also significantly increased, has been shown post-SG to activate hepatic vitamin D receptors (VDR), inducing sulfotransferase expression (mSult2A1/hSULT2A) and promoting the formation of sulfated BAs like CA7S, which subsequently enhances systemic GLP-1 levels and improves glycemia (Chaudhari et al., 2021a,b). Notably, we identified DHLCA, a key oxidative metabolite of LCA, as significantly elevated and correlated with metabolic improvement. DHLCA serves as a potent activator of both FXR and the pregnane X receptor (PXR) (MahmoudianDehkordi et al., 2022). PXR activation contributes to glucose homeostasis by suppressing gluconeogenesis, modulating lipid metabolism, and exerting anti-inflammatory effects (Lv et al., 2022). In our SADI-S cohort, elevated levels of GUDCA have been shown to enhance metabolic health by improving insulin resistance and hyperglycemia (Chen et al., 2023). Although the exact mechanism in our model necessitates further investigation, Chen et al. demonstrated that GUDCA promotes thermogenesis, likely through a gut microbiota-dependent increase in TLCA and subsequent activation of TGR5 in adipose tissue. The collective elevation of these BAs following SADI-S, particularly CDCA, LCA, DHLCA, and GUDCA, suggests a coordinated activation of the FXR, TGR5, VDR, and PXR signaling pathways. These findings may provide a partial molecular foundation for the superior metabolic efficacy of SADI-S relative to SG.

Consistent with the activation of TGR5 and the potential signaling of VDR by elevated BAs, we observed significantly higher post-prandial GLP-1 levels in both the SADI-S and SG groups compared to SO, with a trend favoring SADI-S (Figures 1C,L,M). Notably, the majority of the metabolically beneficial BAs identified (including CDCA, LCA and GUDCA, etc.) demonstrated significant positive correlations with GLP-1 levels (Figure 7). GLP-1, a well-established incretin hormone, stimulates insulin secretion, inhibits glucagon release, delays gastric emptying, and promotes satiety. Its levels are significantly elevated following both RYGB and SG, and this elevation is associated with the metabolic improvements observed after these procedures (Carvalho et al., 2022; Jørgensen et al., 2013; Peterli et al., 2012). Our data strongly support the notion that enhanced GLP-1 secretion likely represents a key downstream effector linking SADI-S and SG-induced BA remodeling—particularly through TGR5 and VDR activation—to improved glycemic control. The superior BA profile induced by SADI-S, characterized by a richness in potent TGR5 agonists such as CDCA and LCA (Figure 4), likely underlies its more pronounced GLP-1 elevation and the consequent metabolic advantage.

Beyond direct receptor signaling, our study underscores the critical interplay between surgically altered BA pools and the reconfigured gut microbiota in driving metabolic benefits. We observed significant shifts in microbial composition at the genus level (Figure 3) and identified compelling correlations between specific bacterial taxa and BA species (Figure 8). Notably, beneficial genera such as *Bifidobacterium* (positively correlated with the FXR/PXR agonist DHLCA) and *unclassified_Muribaculaceae* (positively correlated with GUDCA) were enriched. These genera are prolific producers of short-chain fatty acids (SCFAs) like acetate, propionate, and butyrate (Huang et al., 2023; Meng et al., 2023; Wang et al., 2023). SCFAs themselves stimulate GLP-1 secretion, promote insulin sensitivity, enhance β-cell function, and exert anti-inflammatory effects (Roy et al., 2020), creating a synergistic loop with BA signaling. An intriguing observation was the significant positive correlation between *Escherichia-Shigella* abundance and several metabolically beneficial BAs (CDCA, α, β, ω-MCA, UDCA, GCDCA, GUDCA). While *Escherichia-Shigella* is conventionally associated with inflammation and disease due to its lipopolysaccharide (LPS) production and potential pathogenicity (Jia et al., 2020; Li et al., 2021; Zhou et al., 2025), our findings resonate with those of Huang et al. (2022), who observed an increase in *Escherichia-Shigella* alongside elevated beneficial BAs and improved metabolic parameters following YH1 treatment in patients with T2D. This suggests a context-dependent role. In the post-surgical milieu, elevated anti-inflammatory BAs [e.g., CDCA, UDCA (Ko et al., 2017; Song et al., 2019)] and the dominance of beneficial SCFA producers may mitigate potential negative effects. Furthermore, rapid surgical alterations in bile flow and nutrient delivery could independently promote both *Escherichia-Shigella* proliferation and beneficial BA accumulation without direct causation. Genus-level resolution also precludes distinguishing potentially beneficial strains that have adapted to the new niche.

Collectively, these findings align with the concept of a ‘microbiota-BA-metabolism axis,’ (Chen et al., 2023), suggesting it may be one mechanism through which SADI-S and SG exert their efficacy. Our data suggest that the surgically altered gut environment may orchestrate metabolic improvements through a synergistic interplay of pathways: (1) Direct BA signaling potentiated via receptors (FXR, TGR5, PXR, VDR); (2) Enhanced secretion of GLP-1, likely amplified by BA-mediated TGR5/VDR activation; (3) Microbial community restructuring that favors the production of beneficial metabolites (e.g., short-chain fatty acids); and (4) Reciprocal crosstalk in which the microbiota modulate BA metabolism (e.g., deconjugation, transformation), while BAs reciprocally shape microbial composition and function. This integrated network appears to converge synergistically to drive metabolic benefits. Crucially, by accelerating BA delivery to the distal ileum, SADI-S has the potential to engage this multifaceted axis to a greater extent than SG.

Our study has several limitations. Due to the high cost and increased postoperative mortality rate associated with the T2D rat model, the sample size is limited, which may restrict the statistical power and the generalizability of the research findings. Validation in a larger cohort is required, and ultimately, validation in human patients is necessary. Although 16S rRNA sequencing revealed significant taxonomic shifts and correlations, functional insights into the microbiota, such as metagenomics and metatranscriptomics, are essential for fully deciphering the ‘microbiota-BA-metabolism’ axis. While the correlative data and the existing literature strongly support the involvement of the FXR, TGR5, PXR, and VDR pathways, definitive causal proof requires targeted interventions, including receptor antagonists or agonists and specific BA supplementation or depletion, in future studies. Investigating the temporal dynamics of BA and microbiota changes post-surgery would also provide deeper mechanistic insights. Despite these limitations, our findings robustly highlight distinct BA signatures, particularly the extensive beneficial profile induced by SADI-S, and their intricate links to GLP-1 and microbiota, offering compelling targets such as specific BA species or pathways and key microbial taxa for developing less invasive therapies that mimic the effects of metabolic surgery.

## Conclusion

In conclusion, this study demonstrates that SADI-S and SG induce distinct, surgery-specific BA remodeling in T2D rats, correlating with their efficacy in improving glucose metabolism, lipid profiles, and β-cell integrity. SADI-S resulted in a more substantial elevation of portal vein serum BAs compared to SO (34 species versus 25 in SG), characterized by significant increases in key regulators such as CDCA, LCA, DHLCA, and GUDCA. These alterations in BA levels exhibit strong negative correlations with glycemic markers and triglycerides, alongside positive correlations with GLP-1. Mechanistically, the observed BA signatures are consistent with established pathways (FXR, TGR5, VDR, PXR activation) and GLP-1 enhancement as supported by existing literature. Moreover, the remodeling of gut microbiota and its dynamic interaction with the BA pool establish a cohesive ‘microbiota-BA-metabolism’ axis. Targeting these specific BA pathways or microbial interactions presents promising opportunities for less invasive T2D therapies.

## Data Availability

The 16S rRNA gene sequencing data are deposited in the NCBI repository under the accession number PRJNA1290010. The targeted BA data are available in the MetaboLights repository with the fecal BA dataset accession number MTBLS12715 and the serum BA dataset accession number MTBLS12716.
