# Supplementary material for: SADI-S and SG surgeries induce distinct bile acid profiles linked to improved glucose metabolism via microbiota interactions
Source: Front Microbiol. 2025 Oct 2;16:1579149. doi: 10.3389/fmicb.2025.1579149 (PMC12528041; doi:10.3389/fmicb.2025.1579149)
Supplement: Supplementary file 1 [file Supplementary_file_1.docx]

**16S rRNA Sequencing and Bioinformatic Analysis**

**High-throughput 16S ribosomal RNA gene sequencing**

Total genomic DNA was extracted from 15 samples using the TGuide S96 Magnetic Soil /Stool DNA Kit (Tiangen Biotech (Beijing) Co., Ltd.) according to manufacturer’s instructions. The quality and quantity of the extracted DNA were examined using electrophoresis on a 1.8% agarose gel,and DNA concentration and purity were determined with NanoDrop 2000 UV-Vis spectrophotometer (Thermo Scientific, Wilmington, USA).The full-length 16S rRNA gene were amplified with primer pairs 27F: AGRGTTTGATYNTGGCTCAG and 1492R: TASGGHTACCTTGTTASGACTT.Both the forward and reverse 16S primers were tailed with sample-specific PacBio barcode sequences to allow for multiplexed sequencing. We chose to use barcoded primers because this reduces chimera formation as compared to the alternative protocol in which primers are added in a second PCR reaction. The KOD One PCR Master Mix (TOYOBOLife Science) was used to perform 25 cycles of PCR amplification, with initial denaturation at 95 °C for 2 min, followed by 25 cycles of denaturation at 98 °C for 10 s, annealing at 55 °C for 30 s, and extension at 72 °C for 1 min 30 s, and a final step at 72 °C for 2 min. The total of PCR amplicons were purified with VAHTSTM DNA Clean Beads (Vazyme, Nanjing,China) and quantified using the Qubit dsDNA HS Assay Kit and Qubit 3.0 Fluorometer (Invitrogen, Thermo Fisher Scientific, Oregon, USA). After the individual quantification step, amplicons were pooled in equal amounts. SMRTbell libraries were prepared from the amplified DNA by SMRTbell Express Template Prep Kit 2.0 according to the manufacturer’s instructions (Pacific Biosciences). Purified SMRTbell libraries from the pooled and barcoded samples were sequenced on a PacBio Sequel II platform (Beijing Biomarker Technologies Co., Ltd., Beijing, China)using Sequel II binding kit 2.0. (Please adjust the primer according to the actual amplification sub region)

**Bioinformatic Analysis**

**OTU cluster**

The bioinformatics analysis of this study was performed with the aid of the BMKCloud (http://www.biocloud.net/). The raw reads generated from sequencing were filtered and demultiplexed using the SMRT Link software (version 8.0) with the minPasses ≥5 and minPredictedAccuracy ≥0.9, in order to obtain the circular consensus sequencing (CCS)reads. Subsequently, the lima (version 1.7.0) was employed to assign the CCS sequences to the corresponding samples based on their barcodes. CCS reads containing no primers and those reads beyond the length range (1,200–1,650 bp) were discarded through the recognition of forward and reverse primers and quality filtering using the Cutadapt [[1]](https://dp.biocloud.net/iframe/report/file.html?id=8a828b8291c688c80191e07619450517&path=2b84b33c-7022-11ef-abfd-008cfa5d459c&base=https://api.biocloud.net&token=eyJhbGciOiJSUzI1NiIsInR5cCI6IkpXVCJ9.eyJ0aWQiOiIwOGE2YmVlNy1iN2RjLTQ1NmEtODdkNi1mMWQxYTg4MzJkMDkiLCJ1c2VyX3V1aWQiOiI4YTgzMDBlNTkxYzY3ZmEwMDE5MWUwNmYxZTFjMDMwYyIsInVzZXJfbmFtZSI6InVKMzNLNjMiLCJhdXRob3JpdGllcyI6WyJST0xFX1VTRVIiXSwiZXhwIjoxNzQ5OTcwMTExMTA1LCJyYW1zX2NvdW50IjozMjc2OCwiY3B1c19jb3VudCI6OCwicmVnaW9uIjoiaGciLCJyYW5rX3JvbGUiOiJwc2lsdmVyX3VzZXIiLCJyZWFsX25hbWUiOiLpqazntKAifQ.DrAtbDFr85d-ESjHxhsPIrmjBm1bQh7Ax05bRBdQUPzhPu_4BgKGA3w8a2xfuBPjBjhKWMWLuH287YjJMka1_5cbOL6AvbnkNtjHYE8YSUwasA2coocWZP1wS-AFlkd8ZQGCHfWT4So20luXQWUkQ479DLxy7GV-W9hrRKo-rHl10E0evq31K-Qmcc5bmz8gJ8QOV1v4bIrM9YOkvfpPLtJRGmPTnh5DTqIlu_jyd_Da1FoLnHV_6qP3cEibu5vHoUV4f5BzHRIOmDzEz55tLUqRkDYRBEvufOjcWsl68_6o0jWsn1yw-_54GpNP5Jtc4yI7VrDQYEBrGoUrri5vuw#ref2) (version 2.7)quality control process .The UCHIME algorithm (v8.1)[[2]](https://dp.biocloud.net/iframe/report/file.html?id=8a828b8291c688c80191e07619450517&path=2b84b33c-7022-11ef-abfd-008cfa5d459c&base=https://api.biocloud.net&token=eyJhbGciOiJSUzI1NiIsInR5cCI6IkpXVCJ9.eyJ0aWQiOiIwOGE2YmVlNy1iN2RjLTQ1NmEtODdkNi1mMWQxYTg4MzJkMDkiLCJ1c2VyX3V1aWQiOiI4YTgzMDBlNTkxYzY3ZmEwMDE5MWUwNmYxZTFjMDMwYyIsInVzZXJfbmFtZSI6InVKMzNLNjMiLCJhdXRob3JpdGllcyI6WyJST0xFX1VTRVIiXSwiZXhwIjoxNzQ5OTcwMTExMTA1LCJyYW1zX2NvdW50IjozMjc2OCwiY3B1c19jb3VudCI6OCwicmVnaW9uIjoiaGciLCJyYW5rX3JvbGUiOiJwc2lsdmVyX3VzZXIiLCJyZWFsX25hbWUiOiLpqazntKAifQ.DrAtbDFr85d-ESjHxhsPIrmjBm1bQh7Ax05bRBdQUPzhPu_4BgKGA3w8a2xfuBPjBjhKWMWLuH287YjJMka1_5cbOL6AvbnkNtjHYE8YSUwasA2coocWZP1wS-AFlkd8ZQGCHfWT4So20luXQWUkQ479DLxy7GV-W9hrRKo-rHl10E0evq31K-Qmcc5bmz8gJ8QOV1v4bIrM9YOkvfpPLtJRGmPTnh5DTqIlu_jyd_Da1FoLnHV_6qP3cEibu5vHoUV4f5BzHRIOmDzEz55tLUqRkDYRBEvufOjcWsl68_6o0jWsn1yw-_54GpNP5Jtc4yI7VrDQYEBrGoUrri5vuw#ref4) was used in detecting and removing chimera sequences to obtain the clean reads. Sequences with similarity > 97% were clustered into the same operational taxonomic unit (OTU) by USEARCH[[3]](https://dp.biocloud.net/iframe/report/file.html?id=8a828b8291c688c80191e07619450517&path=2b84b33c-7022-11ef-abfd-008cfa5d459c&base=https://api.biocloud.net&token=eyJhbGciOiJSUzI1NiIsInR5cCI6IkpXVCJ9.eyJ0aWQiOiIwOGE2YmVlNy1iN2RjLTQ1NmEtODdkNi1mMWQxYTg4MzJkMDkiLCJ1c2VyX3V1aWQiOiI4YTgzMDBlNTkxYzY3ZmEwMDE5MWUwNmYxZTFjMDMwYyIsInVzZXJfbmFtZSI6InVKMzNLNjMiLCJhdXRob3JpdGllcyI6WyJST0xFX1VTRVIiXSwiZXhwIjoxNzQ5OTcwMTExMTA1LCJyYW1zX2NvdW50IjozMjc2OCwiY3B1c19jb3VudCI6OCwicmVnaW9uIjoiaGciLCJyYW5rX3JvbGUiOiJwc2lsdmVyX3VzZXIiLCJyZWFsX25hbWUiOiLpqazntKAifQ.DrAtbDFr85d-ESjHxhsPIrmjBm1bQh7Ax05bRBdQUPzhPu_4BgKGA3w8a2xfuBPjBjhKWMWLuH287YjJMka1_5cbOL6AvbnkNtjHYE8YSUwasA2coocWZP1wS-AFlkd8ZQGCHfWT4So20luXQWUkQ479DLxy7GV-W9hrRKo-rHl10E0evq31K-Qmcc5bmz8gJ8QOV1v4bIrM9YOkvfpPLtJRGmPTnh5DTqIlu_jyd_Da1FoLnHV_6qP3cEibu5vHoUV4f5BzHRIOmDzEz55tLUqRkDYRBEvufOjcWsl68_6o0jWsn1yw-_54GpNP5Jtc4yI7VrDQYEBrGoUrri5vuw#ref3) (v10.0), and the OTUs conuts less than 2 in all samples were filtered.

**ASV(dada2)**

Clean reads then were conducted on feature classification to output an ASVs (amplicon sequence variants) by DADA2[[5]](https://dp.biocloud.net/iframe/report/file.html?id=8a828b8291c688c80191e07619450517&path=2b84b33c-7022-11ef-abfd-008cfa5d459c&base=https://api.biocloud.net&token=eyJhbGciOiJSUzI1NiIsInR5cCI6IkpXVCJ9.eyJ0aWQiOiIwOGE2YmVlNy1iN2RjLTQ1NmEtODdkNi1mMWQxYTg4MzJkMDkiLCJ1c2VyX3V1aWQiOiI4YTgzMDBlNTkxYzY3ZmEwMDE5MWUwNmYxZTFjMDMwYyIsInVzZXJfbmFtZSI6InVKMzNLNjMiLCJhdXRob3JpdGllcyI6WyJST0xFX1VTRVIiXSwiZXhwIjoxNzQ5OTcwMTExMTA1LCJyYW1zX2NvdW50IjozMjc2OCwiY3B1c19jb3VudCI6OCwicmVnaW9uIjoiaGciLCJyYW5rX3JvbGUiOiJwc2lsdmVyX3VzZXIiLCJyZWFsX25hbWUiOiLpqazntKAifQ.DrAtbDFr85d-ESjHxhsPIrmjBm1bQh7Ax05bRBdQUPzhPu_4BgKGA3w8a2xfuBPjBjhKWMWLuH287YjJMka1_5cbOL6AvbnkNtjHYE8YSUwasA2coocWZP1wS-AFlkd8ZQGCHfWT4So20luXQWUkQ479DLxy7GV-W9hrRKo-rHl10E0evq31K-Qmcc5bmz8gJ8QOV1v4bIrM9YOkvfpPLtJRGmPTnh5DTqIlu_jyd_Da1FoLnHV_6qP3cEibu5vHoUV4f5BzHRIOmDzEz55tLUqRkDYRBEvufOjcWsl68_6o0jWsn1yw-_54GpNP5Jtc4yI7VrDQYEBrGoUrri5vuw#ref5), and the ASVs conuts less than 2 in all samples were filtered. Taxonomy annotation of the OTUs/ASVs was performed based on the Naive Bayes classifier in QIIME2[[6]](https://dp.biocloud.net/iframe/report/file.html?id=8a828b8291c688c80191e07619450517&path=2b84b33c-7022-11ef-abfd-008cfa5d459c&base=https://api.biocloud.net&token=eyJhbGciOiJSUzI1NiIsInR5cCI6IkpXVCJ9.eyJ0aWQiOiIwOGE2YmVlNy1iN2RjLTQ1NmEtODdkNi1mMWQxYTg4MzJkMDkiLCJ1c2VyX3V1aWQiOiI4YTgzMDBlNTkxYzY3ZmEwMDE5MWUwNmYxZTFjMDMwYyIsInVzZXJfbmFtZSI6InVKMzNLNjMiLCJhdXRob3JpdGllcyI6WyJST0xFX1VTRVIiXSwiZXhwIjoxNzQ5OTcwMTExMTA1LCJyYW1zX2NvdW50IjozMjc2OCwiY3B1c19jb3VudCI6OCwicmVnaW9uIjoiaGciLCJyYW5rX3JvbGUiOiJwc2lsdmVyX3VzZXIiLCJyZWFsX25hbWUiOiLpqazntKAifQ.DrAtbDFr85d-ESjHxhsPIrmjBm1bQh7Ax05bRBdQUPzhPu_4BgKGA3w8a2xfuBPjBjhKWMWLuH287YjJMka1_5cbOL6AvbnkNtjHYE8YSUwasA2coocWZP1wS-AFlkd8ZQGCHfWT4So20luXQWUkQ479DLxy7GV-W9hrRKo-rHl10E0evq31K-Qmcc5bmz8gJ8QOV1v4bIrM9YOkvfpPLtJRGmPTnh5DTqIlu_jyd_Da1FoLnHV_6qP3cEibu5vHoUV4f5BzHRIOmDzEz55tLUqRkDYRBEvufOjcWsl68_6o0jWsn1yw-_54GpNP5Jtc4yI7VrDQYEBrGoUrri5vuw#ref6) using the SILVA database[7] (release 138.1) with a confidence threshold of 70%. The Alpha diversity were calculated and displayed by the QIIME2 and R software, respectively. Beta diversity was determined to evaluate the degree of similarity of microbial communities from different samples using QIIME. Principal coordinate analysis (PCoA), heatmaps, UPGMA and nonmetric multidimensional scaling (NMDS) were used to analyze the beta diversity. Furthermore, we employed Linear Discriminant Analysis (LDA) effect size (LEfSe[[8]](https://dp.biocloud.net/iframe/report/file.html?id=8a828b8291c688c80191e07619450517&path=2b84b33c-7022-11ef-abfd-008cfa5d459c&base=https://api.biocloud.net&token=eyJhbGciOiJSUzI1NiIsInR5cCI6IkpXVCJ9.eyJ0aWQiOiIwOGE2YmVlNy1iN2RjLTQ1NmEtODdkNi1mMWQxYTg4MzJkMDkiLCJ1c2VyX3V1aWQiOiI4YTgzMDBlNTkxYzY3ZmEwMDE5MWUwNmYxZTFjMDMwYyIsInVzZXJfbmFtZSI6InVKMzNLNjMiLCJhdXRob3JpdGllcyI6WyJST0xFX1VTRVIiXSwiZXhwIjoxNzQ5OTcwMTExMTA1LCJyYW1zX2NvdW50IjozMjc2OCwiY3B1c19jb3VudCI6OCwicmVnaW9uIjoiaGciLCJyYW5rX3JvbGUiOiJwc2lsdmVyX3VzZXIiLCJyZWFsX25hbWUiOiLpqazntKAifQ.DrAtbDFr85d-ESjHxhsPIrmjBm1bQh7Ax05bRBdQUPzhPu_4BgKGA3w8a2xfuBPjBjhKWMWLuH287YjJMka1_5cbOL6AvbnkNtjHYE8YSUwasA2coocWZP1wS-AFlkd8ZQGCHfWT4So20luXQWUkQ479DLxy7GV-W9hrRKo-rHl10E0evq31K-Qmcc5bmz8gJ8QOV1v4bIrM9YOkvfpPLtJRGmPTnh5DTqIlu_jyd_Da1FoLnHV_6qP3cEibu5vHoUV4f5BzHRIOmDzEz55tLUqRkDYRBEvufOjcWsl68_6o0jWsn1yw-_54GpNP5Jtc4yI7VrDQYEBrGoUrri5vuw#ref8)) to test the significant taxonomic difference among group. A logarithmic LDA score of 4.0 was set as the threshold for discriminative features. To explore the dissimilarities of the microbiome among different factors, a redundancy analysis (RDA) were performed in R using the package vegan.

**Table 1.List of Main Reagents**

| **Name** | **Source** |
| --- | --- |
| TGuide S96 Magnetic Soil/Stool DNA Kit | Tiangen Biotech (Beijing) Co., Ltd. |
| KOD One PCR Master Mix | TOYOBO Life Science |
| VAHTSTM DNA Clean Beads | Vazyme, Nanjing, China |
| Qubit dsDNA HS Assay Kit | Invitrogen, Thermo Fisher Scientific, Oregon, USA |
| SMRTbell Express Template Prep Kit 2.0 | Pacific Biosciences |
| Sequel II binding kit 2.0 | Pacific Biosciences |

**Table 2. List of main software**

| **Name** | **Source** | **Version/Batch Number** |
| --- | --- | --- |
| SMRT Link | Pacific Biosciences | v8.0 |
| lima | - | v1.7.0 |
| Cutadapt | - | v2.7 |
| UCHIME | - | v8.1 |
| USEARCH | - | v10.0 |
| DADA2 | - | - |
| QIIME2 | - | - |
| R (vegan package) | - | - |

**Reference**

[1][Bolger A M, Lohse M, Usadel B. Trimmomatic: a flexible trimmer for Illumina sequence data[J]. Bioinformatics, 2014, 30(15): 2114-2120.](https://doi.org/10.1093/bioinformatics/btu170)

[2][Martin M. Cutadapt removes adapter sequences from high-throughput sequencing reads[J]. EMBnet. journal, 2011, 17(1): 10-12.](https://doi.org/10.14806/ej.17.1.200)

[3][Edgar R C. UPARSE: highly accurate OTU sequences from microbial amplicon reads[J]. Nature methods, 2013, 10(10): 996.](http://www.drive5.com/usearch/)

[4][Edgar R C, Haas B J, Clemente J C, et al. UCHIME improves sensitivity and speed of chimera detection[J]. Bioinformatics, 2011, 27(16): 2194-2200.](https://doi.org/10.1093/bioinformatics/btr381)

[5][Callahan B J, McMurdie P J, Rosen M J, et al. DADA2: High-resolution sample inference from Illumina amplicon data[J]. Nature methods, 2016, 13(7): 581-583.](https://doi.org/10.1038/nmeth.3869)

[6][Bolyen E, Rideout J R, Dillon M R, et al. Reproducible, interactive, scalable and extensible microbiome data science using QIIME 2[J]. Nature biotechnology, 2019, 37(8): 852-857.](https://doi.org/10.1038/s41587-019-0209-9)

[7][Quast C, Pruesse E, Yilmaz P, et al. The SILVA ribosomal RNA gene database project: improved data processing and web-based tools[J]. Nucleic acids research, 2012, 41(D1): D590-D596.](https://doi.org/10.1093/nar/gks1219)

[8][Segata N, Izard J, Waldron L, et al. Metagenomic biomarker discovery and explanation[J]. Genome biology, 2011, 12(6): 1-18.](https://doi.org/10.1186/gb-2011-12-6-r60)
