# Supplementary material for: SADI-S and SG surgeries induce distinct bile acid profiles linked to improved glucose metabolism via microbiota interactions
Source: Front Microbiol. 2025 Oct 2;16:1579149. doi: 10.3389/fmicb.2025.1579149 (PMC12528041; doi:10.3389/fmicb.2025.1579149)
Supplement: Supplementary file 2 [file Supplementary_file_2.docx]

**Bile Acid Quantification**

**1. Experimental Methods**

****1.1. Sample Preparation and Metabolite Extraction****
Frozen samples were thawed on ice. Approximately 50 mg of each tissue sample (wet weight) was accurately weighed into a pre-chilled 2 mL microcentrifuge tube containing stainless steel grinding beads. One milliliter of ice-cold extraction solvent (acetonitrile: methanol: water, 2:2:1, v/v/v) containing 0.1% (v/v) formic acid and a mixture of deuterated bile acid internal standards (final concentration 50 nmol/L for each) was added. The mixture was immediately vortexed vigorously for 30 seconds to ensure homogenization and initiate protein precipitation. Samples were then subjected to mechanical homogenization at 25 Hz for 10 minutes using a bead mill homogenizer, followed by vortexing for 3 minutes and incubation on ice for 30 minutes to facilitate metabolite extraction. After incubation, samples were centrifuged at 12,000 × g and 4 °C for 10 minutes to pellet insoluble debris. The resulting supernatant (500 μL) was carefully transferred to a new microcentrifuge tube, filtered through a 0.22 μm nylon membrane filter, and transferred to a labeled LC-MS vial for subsequent analysis. All samples were maintained at 4 °C in the autosampler during the analytical sequence.

****1.2. Standard and Calibration Solution Preparation****
Stock solutions of individual bile acid reference standards were prepared at a concentration of 5 mg/mL in methanol and stored at -80 °C. A mixed intermediate standard solution (50 μg/mL for each analyte) was prepared in methanol by appropriate dilution of the stock solutions. A series of calibration standard solutions, spanning the expected physiological concentration range in the biological matrix, were prepared by serial dilution of the intermediate standard solution in the initial extraction solvent (acetonitrile: methanol: water, 2:2:1, v/v/v, containing 0.1% formic acid and the same concentration of internal standards as used for samples). Quality control (QC) samples at low, medium, and high concentrations within the calibration range were prepared similarly from an independent weighing of standards.

****1.3. UHPLC-MRM-MS/MS Analysis****
Chromatographic separation was performed on a Waters ACQUITY I-Class UHPLC system (Waters Corporation, Milford, MA, USA) equipped with an ACQUITY UPLC BEH C18 column (100 mm × 2.1 mm i.d., 1.7 μm particle size; Waters) maintained at 35 °C. The mobile phase consisted of (A) 0.1% (v/v) formic acid and 5 mM ammonium acetate in water and (B) acetonitrile: methanol (3:1, v/v). The following gradient elution program was used at a flow rate of 0.4 mL/min: 0-1 min, 20% B; 1-6 min, 20-50% B; 6-8 min, 50-70% B; 8-10 min, 70-95% B; 10-12 min, 95% B; 12-12.1 min, 95-20% B; followed by re-equilibration at 20% B for 3.9 min (total run time 16 min). The autosampler temperature was set at 10 °C, and the injection volume was 2 μL.
Mass spectrometric detection was carried out using a SCIEX QTRAP 6500+ triple quadrupole mass spectrometer (SCIEX, Framingham, MA, USA) equipped with an IonDrive Turbo V electrospray ionization (ESI) source operating in negative ion mode. Source parameters were optimized as follows: curtain gas (CUR) = 35 psi, ion spray voltage (IS) = -4500 V, temperature (TEM) = 550 °C, ion source gas 1 (GS1) = 50 psi, ion source gas 2 (GS2) = 55 psi. Nitrogen was used for all gas supplies. Instrument control and data acquisition were performed using SCIEX Analyst Work Station Software (Version 1.7.2).
Due to the inherent difficulty in generating abundant characteristic fragment ions for many unconjugated bile acids under conventional collision-induced dissociation (CID) conditions, quantification was primarily achieved using a pseudo-Multiple Reaction Monitoring (pseudo-MRM) approach. Specifically, for unconjugated bile acids lacking dominant fragments, the transition monitored was the precursor ion ([M-H]⁻) to the precursor ion itself (Q1 = Q3 = m/z of [M-H]⁻). For conjugated bile acids (glycine- and taurine-conjugates) and unconjugated species producing quantifiable fragments, the most intense and specific fragment ions were selected for quantification. Additional qualifying transitions were monitored for all analytes to confirm identity based on retention time and ion ratio consistency. Optimal compound-dependent parameters (declustering potential, entrance potential, collision energy, and collision cell exit potential) for each MRM transition were determined by direct infusion and flow injection analysis of individual standard solutions. Data processing was performed using Sciex OS software (Version 2.0.1).

****1.4. Calibration Curves and Quantification****
Calibration curves were constructed for each analyte by plotting the peak area ratio of the analyte to its corresponding deuterated internal standard (y-axis) against the nominal concentration of the analyte (x-axis, ng/mL). Weighted (1/x) least squares linear regression was applied to establish the best-fit line. Calibration points demonstrating accuracy (calculated concentration / nominal concentration × 100%) outside the acceptable range of 80-120% were excluded from the curve fitting. The correlation coefficient (R) for all calibration curves exceeded 0.995, indicating excellent linearity. Quantification of bile acids in samples was performed by interpolating the analyte/internal standard peak area ratio against the respective calibration curve.

****1.5. Method Validation: Sensitivity, Precision, and Accuracy****
Method sensitivity was evaluated by determining the lower limit of detection (LLOD) and lower limit of quantitation (LLOQ). The LLOD was defined as the lowest concentration yielding a signal-to-noise ratio (S/N) ≥ 3, while the LLOQ was defined as the lowest concentration yielding an S/N ≥ 10 and meeting accuracy (80-120%) and precision (relative standard deviation, RSD ≤ 20%) criteria, as per US FDA bioanalytical method validation guidelines. These were established by analyzing serially diluted calibration solutions (dilution factor 2).
Precision (intra-assay) was assessed as the relative standard deviation (RSD, %) by analyzing six replicates of QC samples at low, medium, and high concentrations within the same analytical batch. Accuracy was evaluated as the percent recovery, calculated as (mean measured concentration / nominal spiked concentration) × 100%. The method demonstrated acceptable precision (RSD < 6%) and accuracy (recoveries between 91.2% and 113.7%) across the three QC levels for all targeted bile acids.
Quality control measures included the analysis of solvent blanks, calibration standards, QC samples at multiple levels, and pooled biological quality control (PBQC) samples interspersed throughout the analytical sequence to monitor system stability and performance. Chromatographic performance was assessed by evaluating peak symmetry, resolution of critical pairs, and retention time stability.

**Table 1.List of main reagents**

| **Name** | **CAS Number** | **Purity Level** | **Brand** |
| --- | --- | --- | --- |
| Methanol | 67-56-1 | LC-MS Grade | Merck Chemical Technology (Shanghai) Co., Ltd. |
| Acetonitrile | 75-05-8 | LC-MS Grade | Merck Chemical Technology (Shanghai) Co., Ltd. |
| Formic acid  **Table 2. Information on target bile acid analytes** | 64-18-6 | LC-MS Grade | Thermo Fisher Scientific (China) Co., Ltd. |

| **Cpd Name** | **CAS** | **KEGG.ID** | **M.W.** | **Formula** | **Polarity** | **Prec Ion** | **Quantifier Ion** | **Qualifier Ion** |
| --- | --- | --- | --- | --- | --- | --- | --- | --- |
| Dehydrolithocholic acid | 1553-56-6 | - | 374.6 | C24H38O3 | Negative | 373.2 | 373.2 | - |
| Isolithocholic acid | 1534-35-6 | C17658 | 376.6 | C24H40O3 | Negative | 375.2 | 375.2 | - |
| Lithocholic acid | 434-13-9 | C03990 | 376.6 | C24H40O3 | Negative | 375.1 | 375.1 | - |
| 23-Nordeoxycholic acid | 53608-86-9 | - | 378.6 | C23H38O4 | Negative | 377.4 | 377.4 | 331.4 |
| 7-Ketolithocholic acid | 4651-67-6 | - | 390.6 | C24H38O4 | Negative | 389.4 | 389.4 | - |
| 12-Ketolithocholic acid | 5130-29-0 | - | 390.6 | C24H38O4 | Negative | 389.3 | 389.3 | - |
| Apocholic acid | 641-81-6 | C15375 | 390.6 | C24H38O4 | Negative | 389.4 | 389.4 | - |
| Ursodeoxycholic acid | 128-13-2 | C07880 | 392.6 | C24H40O4 | Negative | 391.3 | 391.3 | - |
| Hyodeoxycholic acid | 83-49-8 | - | 392.6 | C24H40O4 | Negative | 391.5 | 391.5 | - |
| Chenodeoxycholic acid | 474-25-9 | C02528 | 392.6 | C24H40O4 | Negative | 391.4 | 391.4 | - |
| Deoxycholic acid | 83-44-3 | C04483 | 392.6 | C24H40O4 | Negative | 391.3 | 391.3 | 343.2 |
| Isodeoxycholic acid | 566-17-6 | C17661 | 392.6 | C24H40O4 | Negative | 391.3 | 391.3 | 345.2 |
| Dehydrocholic acid | 81-23-2 | C13154 | 402.5 | C24H34O5 | Negative | 401.2 | 401.2 | 331.3 |
| 7,12-Diketolithocholic acid | 517-33-9 | - | 404.5 | C24H36O5 | Negative | 403.2 | 403.2 | 385.3 |
| 7-Ketodeoxycholic acid | 911-40-0 | - | 406.6 | C24H38O5 | Negative | 405.5 | 405.5 | - |
| 12-Dehydrocholic acid | 2458-08-4 | - | 406.6 | C24H38O5 | Negative | 405.5 | 405.5 | - |
| 3-Dehydrocholic acid | 2304-89-4 | - | 406.6 | C24H38O5 | Negative | 405.1 | 405.1 | 289 |
| Ursocholic acid | 2955-27-3 | C17644 | 408.6 | C24H40O5 | Negative | 407.5 | 407.5 | - |
| α-Muricholic acid | 2393-58-0 | C17647 | 408.6 | C24H40O5 | Negative | 407.4 | 407.4 | 405.1 |
| β-Muricholic acid | 2393-59-1 | C17726 | 408.6 | C24H40O5 | Negative | 407.5 | 407.5 | - |
| Hyocholic acid | 547-75-1 | C17649 | 408.6 | C24H40O5 | Negative | 407.4 | 407.4 | - |
| Allocholic acid | 2464-18-8 | C17737 | 408.6 | C24H40O5 | Negative | 407.2 | 407.2 | 361.1 |
| Cholic acid | 81-25-4 | C00695 | 408.6 | C24H40O5 | Negative | 407.3 | 407.3 | 343.4 |
| Glycolithocholic acid | 474-74-8 | C15557 | 433.6 | C26H43NO4 | Negative | 432.3 | 432.3 | 388.5 |
| Glycoursodeoxycholic acid | 64480-66-6 | - | 449.6 | C26H43NO5 | Negative | 448.2 | 448.2 | 386.3 |
| Glycohyodeoxycholic acid | 13042-33-6 | - | 449.6 | C26H43NO5 | Negative | 448.4 | 448.4 | 386.4 |
| Glycodeoxycholic acid | 360-65-6 | C05464 | 449.6 | C26H43NO5 | Negative | 448.3 | 448.3 | 404.2 |
| Glycodehydrocholic acid | 3415-45-0 | - | 459.6 | C26H37NO6 | Negative | 458.3 | 458.3 | 348.3 |
| Glycocholic acid | 475-31-0 | C01921 | 465.6 | C26H43NO6 | Negative | 464.3 | 464.3 | 402.4 |
| Taurolithocholic acid | 6042-32-6 | C02592 | 483.7 | C26H45NO5S | Negative | 482.4 | 482.4 | 80 |
| Tauroursodeoxycholic acid | 14605-22-2 | C16868 | 499.7 | C26H45NO6S | Negative | 498.2 | 498.2 | 106.9 |
| Taurohyodeoxycholic acid | 2958-04-5 | - | 499.7 | C26H45NO6S | Negative | 498.4 | 498.4 | 107 |
| Taurochenodeoxycholic acid | 516-35-8 | C05465 | 499.7 | C26H45NO6S | Negative | 498.3 | 498.3 | 124 |
| Taurodeoxycholic acid | 516-50-7 | C05463 | 499.7 | C26H45NO6S | Negative | 498.2 | 498.2 | 124 |
| Tauro α-Muricholic acid | 25613-05-2 | - | 515.7 | C26H45NO7S | Negative | 514.2 | 514.2 | 124 |
| Taurocholic acid | 81-24-3 | C05122 | 515.7 | C26H45NO7S | Negative | 514.3 | 514.3 | 124 |
| Isoallolithocholic acid | 2276-93-9 | - | 376.6 | C24H40O3 | Negative | 375.4 | 375.4 | - |
| Murideoxycholic acid | 668-49-5 | C15515 | 392.6 | C24H40O4 | Negative | 391.3 | 391.3 | - |
| Isoursodeoxycholic acid | 78919-26-3 | C17662 | 392.6 | C24H40O4 | Negative | 391.2 | 391.2 | - |
| Isohyodeoxycholic acid | 570-84-3 | - | 392.6 | C24H40O4 | Negative | 391.4 | 391.4 | - |
| 3-Epideoxycholic acid | 570-63-8 | - | 392.6 | C24H40O4 | Negative | 391.1 | 391.1 | - |
| Nor Cholic Acid | 60696-62-0 | - | 394.5 | C23H38O5 | Negative | 393.3 | 393.3 | 331.3 |
| 3β-Cholic Acid | 3338-16-7 | - | 408.6 | C24H40O5 | Negative | 407.2 | 407.2 | - |
| ω-Muricholic Acid | 6830-03-1 | - | 408.6 | C24H40O5 | Negative | 407.2 | 407.2 | - |
| Glycochenodeoxycholic acid | 640-79-9 | C05466 | 449.6 | C26H43NO5 | Negative | 448.4 | 448.4 | 386.3 |
| Lithocholic Acid-3-Sulfate | 64936-81-8 | - | 518.6 | C24H40Na2O7S | Negative | 455.2 | 455.2 | 375.3 |
| Glycohyocholic acid | 32747-08-3 | - | 465.6 | C26H43NO6 | Negative | 464.3 | 464.3 | 354.4 |
| Tauro β-Muricholic acid | 25696-60-0 | - | 515.7 | C26H45NO7S | Negative | 514.1 | 514.1 | - |
| Taurohyocholic acid | 32747-07-2 | C15516 | 515.7 | C26H45NO7S | Negative | 514.1 | 514.1 | 124 |
| Chenodeoxycholic acid-3-β-D-Glucuronide | 58814-71-4 | - | 568.7 | C30H48O10 | Negative | 567.3 | 567.3 | 391.3 |

**Table 3. List of main instruments**

| **Instrument** | **Model** | **Brand** |
| --- | --- | --- |
| Ultra-high Performance LC | Waters ACQUITY I-Class | Waters Corporation (Shanghai) Co., Ltd. |
| Mass Spectrometer | SCIEX QTRAP 6500+ | SCIEX |
| High-Speed Refrigerated Centrifuge | GL0650R | Monad |
| Multi-Channel Vortex Mixer | TL2020 | Beijing Dinghaoyuan Technology Co., Ltd. |
| Balance | QUINTIX244-1CN | Sartorius |

**Table 4. List of main software**

| **Software Name** | **Version** | **Manufacturer** | **Purpose** |
| --- | --- | --- | --- |
| SCIEX Analyst Work Station Software | 1.7.2 | SCIEX, Framingham, MA, USA | Instrument control and data acquisition |
| Sciex OS Software | 2.0.1 | SCIEX, Framingham, MA, USA | Data processing and analysis |
