# Supplementary material for: SADI-S and SG surgeries induce distinct bile acid profiles linked to improved glucose metabolism via microbiota interactions
Source: Front Microbiol. 2025 Oct 2;16:1579149. doi: 10.3389/fmicb.2025.1579149 (PMC12528041; doi:10.3389/fmicb.2025.1579149)
Supplement: Supplementary file 3 [file Supplementary_file_3.docx]

| **Name** | **Abbreviation** |
| --- | --- |
| Chenodeoxycholic acid | CDCA |
| α-Muricholic acid | α-MCA |
| β-Muricholic acid | β-MCA |
| Cholic acid | CA |
| Dehydrolithocholic acid | DHLCA |
| Isolithocholic acid | iso-LCA |
| Lithocholic acid | LCA |
| 23-Nordeoxycholic acid | 23-norDCA |
| 7-Ketolithocholic acid | 7-ketoLCA |
| 12-Ketolithocholic acid | 12-ketoLCA |
| Apocholic acid | Apocholic acid |
| Ursodeoxycholic acid | UDCA |
| Hyodeoxycholic acid | HDCA |
| Deoxycholic acid | DCA |
| Isodeoxycholic acid | iso-DCA |
| Dehydrocholic acid | DHCA |
| 7,12-Diketolithocholic acid | 7,12-diketoLCA |
| 7-Ketodeoxycholic acid | 7-ketoDCA |
| 12-Dehydrocholic acid | 12-DHCA |
| 3-Dehydrocholic acid | 3-DHCA |
| Ursocholic acid | UCA |
| Hyocholic acid | HCA |
| Allocholic acid | ACA |
| Isoallolithocholic acid | isoallo-LCA |
| Murideoxycholic acid | MDCA |
| Isoursodeoxycholic acid | iso-UDCA |
| Isohyodeoxycholic acid | iso-HDCA |
| 3-Epideoxycholic acid | 3-EDCA |
| Nor Cholic Acid | Nor CA |
| 3β-Cholic Acid | 3β-CA |
| ω-Muricholic Acid | ωMCA |
| Lithocholic Acid-3-Sulfate | LCA-3-S |
| Chenodeoxycholic acid-3-β-D-Glucuronide | CDCA-3-β-D-G |
| Glycocholic acid | GCA |
| Taurochenodeoxycholic acid | TCDCA |
| Tauro α-Muricholic acid | TαMCA |
| Taurocholic acid | TCA |
| Glycochenodeoxycholic acid | GCDCA |
| Tauro β-Muricholic acid | TβMCA |
| Glycolithocholic acid | GLCA |
| Glycoursodeoxycholic acid | GUDCA |
| Glycohyodeoxycholic acid | GHDCA |
| Glycodeoxycholic acid | GDCA |
| Glycodehydrocholic acid | GDHCA |
| Taurolithocholic acid | TLCA |
| Tauroursodeoxycholic acid | TUDCA |
| Taurohyodeoxycholic acid | THDCA |
| Taurodeoxycholic acid | TDCA |
| Glycohyocholic acid | GHCA |
| Taurohyocholic acid | THCA |
